# Supplementary material for: Density-Dependent Differentiation of Tonsil-Derived Mesenchymal Stem Cells into Parathyroid-Hormone-Releasing Cells
Source: Int J Mol Sci. 2022 Jan 10;23(2):715. doi: 10.3390/ijms23020715 (PMC8775366; doi:10.3390/ijms23020715)
Supplement: Supplementary file 1 [file ijms-23-00715-s001.zip › ijms-1504399-supplementary.pdf]

Supplemental Table S1. Top 20 most significantly upregulated expressed genes of the T-MSC-PTHCs.

Genes	transcript_id	Description	Fold change of T-MSC-PTHC (per T-MSC)
MBD6	NM_052897	methyl-CpG binding domain protein 6	3.47
PSG4	NM_002780	pregnancy specific beta-1-glycoprotein 4	2.974
WNT2	NR_024047	Wnt family member 2	2.881
EFNB2	NM_004093	ephrin B2	2.769
LOC284344	NR_033888	uncharacterized LOC284344	2.738
PSG5	NM_001130014	pregnancy specific beta-1-glycoprotein 5	2.691
TNFSF18	NM_005092	tumor necrosis factor superfamily member 18	2.67
LPAR1	NM_057159	lysophosphatidic acid receptor 1	2.591
ADRA1B	NM_000679	adrenoceptor alpha 1B	2.555
CLSTN2	NM_022131	calsyntenin 2	2.445
LMCD1	NM_001278235	LIM and cysteine rich domains 1	2.422
ZBTB41	NM_194314	zinc finger and BTB domain containing 41	2.359
DNAJC6	NM_001256865	DnaJ heat shock protein family (Hsp40) member C6	2.342
PRKAB1	NM_006253	protein kinase AMP-activated non-catalytic subunit beta 1	2.321
ZNF672	NM_024836	zinc finger protein 672	2.315
CPA4	NM_016352	carboxypeptidase A4	2.265
OXTR	NM_000916	oxytocin receptor	2.263
MBD7	NM_000088	collagen type I alpha 1	2.202
PSG5	NM_003186	transgelin	2.186
WNT3	NM_000053	ATPase copper transporting beta	2.183

Supplemental Table S2. Top 20 most significantly downregulated expressed genes of the T-MSC-PTHCs.

Genes	transcript_id	Description	Fold change of T-MSC-PTHC (per T-MSC)
NPTX1	NM_002522	neuronal pentraxin 1	0.310
TMEFF2	NM_016192	transmembrane protein with EGF like and two follistatin like domains 2	0.365
PIGG	NM_001289051	phosphatidylinositol glycan anchor biosynthesis class G	0.366
PLXDC1	NM_020405	plexin domain containing 1	0.394
HIST1H1B	NM_005322	histone cluster 1, H1b	0.398
ANXA2	NM_004039	annexin A2	0.410
TMEM100	NM_001099640	transmembrane protein 100	0.418
MOB3B	NM_024761	MOB kinase activator 3B	0.421
LOC286437	NR_039980	uncharacterized LOC286437	0.429
RRN3P1	NR_003370	RRN3 homolog, RNA polymerase I transcription factor pseudogene 1	0.450
SCARA5	NM_173833	scavenger receptor class A member 5	0.458
KIAA2013	NM_138346	KIAA2013	0.459
CH25H	NM_003956	cholesterol 25-hydroxylase	0.463
MAMSTR	NM_001297753	MEF2 activating motif and SAP domain containing transcriptional regulator	0.464
KITLG	NM_003994	KIT ligand	0.466
FAM216B	NM_182508	family with sequence similarity 216 member B	0.477
PIK3C2A	NM_002645	phosphatidylinositol-4-phosphate 3-kinase catalytic subunit type 2 alpha	0.482
GAP43	NM_002045	growth associated protein 43	0.482
COL14A1	NM_021110	collagen type XIV alpha 1	0.490
KIAA1324L	NM_002522	KIAA1324-like	0.501
